# Crystal structures of two tandem malectin-like receptor kinases involved in plant reproduction

**DOI:** 10.1107/S205979831800774X

**Published:** 2018-06-27

**Authors:** Steven Moussu, Sebastian Augustin, Andra-Octavia Roman, Caroline Broyart, Julia Santiago

**Affiliations:** a The Plant Signaling Mechanisms Laboratory, Department of Plant Molecular Biology, University of Lausanne, 1015 Lausanne, Switzerland

**Keywords:** malectin-like receptor kinases, plant reproductive signalling, cell-wall signalling, membrane signalling, *Arabidopsis*, cell signalling, membrane receptors, receptor kinases, *Catharanthus roseus*

## Abstract

Complex cell-to-cell communication between the male pollen tube and the female reproductive organs is required for plant fertilization. A family of *Catharanthus roseus* receptor kinase 1-like (*Cr*RLK1L) membrane receptors has been genetically implicated in this process. Here, crystal structures of the *Cr*RLK1Ls ANXUR1 and ANXUR2 are reported at 1.48 and 1.1 Å resolution, respectively. The structures reveal a novel arrangement of two malectin-like domains connected by a short β-hairpin linker and stabilized by calcium ions. The canonical carbohydrate-interaction surfaces of related animal and bacterial carbohydrate-binding modules are not conserved in plant *Cr*RLK1Ls. In line with this, the binding of chemically diverse oligosaccharides to ANXUR1 and HERCULES1 could not be detected. Instead, *Cr*RLK1Ls have evolved a protein–protein interface between their malectin domains which forms a deep cleft lined by highly conserved aromatic and polar residues. Analysis of the glycosylation patterns of different *Cr*RLK1Ls and their oligomeric states suggests that this cleft could resemble a binding site for a ligand required for receptor activation of *Cr*RLK1Ls.

## Abstract 2

The biochemical and crystallographic characterization of different *Catharanthus roseus* receptor kinase 1-like membrane receptors is reported as a first step to understand cell-wall sensing and signalling mechanisms in plants.

## 1. Introduction   

Plant cells are surrounded by a dynamic, carbohydrate-rich cell wall which is constantly remodelled to enable coordinated growth and development, and serves as a link to the outside world (Ringli, 2010 ▸). To sense and integrate environmental and internal signals, plants have evolved a set of membrane receptor kinases (RKs), the extracellular domains of which face the cell-wall compartment (Shiu & Bleecker, 2001 ▸, 2003 ▸). Members of the plant-specific *Catharanthus roseus* receptor kinase 1-like (*Cr*RLK1L) family are key players in monitoring the cell-wall status and regulating cell expansion (Voxeur & Höfte, 2016 ▸; Wolf *et al.*, 2012 ▸; Boisson-Dernier *et al.*, 2011 ▸). Of the 17 members in *Arabidopsis*, ten have been functionally characterized: FERONIA (FER), ANXUR1 (ANX1), ANXUR2 (ANX2), BUDDHA PAPER SEAL 1/2 (BUPS1/2), HERCULES1 (HERK1), HERCULES2 (HERK2), [Ca^2+^]cyt-associated protein kinase 1 (CAP1/ERULUS), THESEUS1 (THE1) and CURVY1 (CVY1) (Nissen *et al.*, 2016 ▸). FER, ANX1/2 and BUPS1/2 are involved in cell-communication events regulating plant fertilization, with FER acting in the female gametophyte and ANX1/2 and BUPS1/2 in the male gametophyte, the pollen. Loss of FER function impairs pollen-tube reception (Escobar-Restrepo *et al.*, 2007 ▸), whereas ANX1/2, together with BUPS1/2, helps to maintain pollen-tube integrity during polarized tip growth, assuring fertilization (Boisson-Dernier *et al.*, 2009 ▸; Ge *et al.*, 2017 ▸; Miyazaki *et al.*, 2009 ▸). FER, THE1, HERK1 and HERK2 have been implicated in regulating cell expansion during vegetative growth (Guo, Ye *et al.*, 2009 ▸; Guo, Li *et al.*, 2009 ▸), with FER having additional roles in plant immunity (Keinath *et al.*, 2010 ▸; Kessler *et al.*, 2010 ▸; Stegmann *et al.*, 2017 ▸). THE1 has been described to be able to sense structural changes in the cell wall and to regulate lignin accumulation in cellulose-deficient mutants (Hématy *et al.*, 2007 ▸). Recent studies have also linked a member of this receptor family to the control of cell morphogenesis and cytoskeleton assembly (Gachomo *et al.*, 2014 ▸).

*Cr*RLK1L family members localize to the plasma membrane (Hématy *et al.*, 2007 ▸) and are composed of a cytoplasmic kinase domain, a single transmembrane helix and a variable ligand-binding ectodomain. The extracellular domain shows weak sequence homology to animal malectin carbohydrate-binding domains (Boisson-Dernier *et al.*, 2011 ▸; Schallus *et al.*, 2008 ▸). A domain-swap analysis of several members of the family suggested that the signalling specificity of *Cr*RLK1Ls is encoded in their extracellular domains. In contrast, their intracellular kinase domain can be interchanged, suggesting that this receptor family shares common downstream signalling components (Kessler *et al.*, 2015 ▸). In the case of FER, the connecting transmembrane domain is also involved in regulating receptor activation (Minkoff *et al.*, 2017 ▸). Whether the extracellular domains of *Cr*RLK1Ls actually form ligand-binding domains for carbohydrate and/or protein ligands still remains to be characterized at the molecular level. To date, several secreted ∼40 amino-acid peptides of the RAPID ALKALINIZATION FACTOR (RALF) family have been proposed as ligands for FER, ANX1/2 and BUPS1/2 (Haruta *et al.*, 2014 ▸; Stegmann *et al.*, 2017 ▸; Ge *et al.*, 2017 ▸; Pearce *et al.*, 2001 ▸), but *Cr*RLK1Ls have also been speculated to interact directly with cell-wall components (Li *et al.*, 2016 ▸; Nissen *et al.*, 2016 ▸; Voxeur & Höfte, 2016 ▸). Biochemical analysis of *Cr*RLK1Ls has been hampered by difficulties in producing active, recombinant protein samples on one hand and by the overwhelming chemical complexity of the plant cell wall on the other hand. Here, we report the expression, purification and biochemical and crystallographic characterization of different *Cr*RLK1Ls as a first step to understand cell-wall sensing and signalling mechanisms in plants.

## 2. Materials and methods   

### 2.1. Protein expression and purification   

Codon-optimized synthetic genes for expression in *Spodoptera frugiperda* (Invitrogen GeneArt, Germany) coding for the *Arabidopsis thaliana* ANX1 (residues 1–429), ANX2 (residues 1–431), HERK1 (residues 1–405) and THE1 (residues 1–403) ectodomains were cloned into a modified pFastBac vector (Geneva Biotech), providing a *Tobacco etch virus* protease (TEV)-cleavable C-terminal StrepII-9×His tag. For protein expression, *Trichoplusia ni* Tnao38 cells (Hashimoto *et al.*, 2012 ▸) were infected with a multiplicity of infection (MOI) of 2 and incubated for 3 d at 22°C and 110 rev min^−1^. The secreted ectodomains were purified from the supernatant by sequential Ni^2+^ (HisTrap Excel equilibrated in 25 m*M* potassium phosphate pH 7.8, 500 m*M* NaCl; GE Healthcare) and StrepII (Strep-Tactin Superflow high capacity equilibrated in 25 m*M* Tris pH 8.0, 250 m*M* NaCl, 1 m*M* EDTA; IBA) affinity chromatography. The proteins were further purified by size-exclusion chromatography on a Superdex 200 Increase 10/300 GL column (GE Healthcare) equilibrated in 20 m*M* sodium citrate pH 5.0, 150 m*M* NaCl. For crystallization and biochemical experiments, proteins were concentrated using Amicon Ultra concentrators (molecular-weight cutoff 10 000; Millipore). Proteins were analyzed for purity and structural integrity by SDS–PAGE and mass spectrometry. The molecular weights of the purified, heterogeneously glycosylated proteins were determined to be ∼58 kDa (ANX1 ectodomain), ∼54 kDa (ANX2), ∼53.5 kDa (HERK1) and ∼63 kDa (THE1).

### 2.2. Crystallization and data collection   

Crystals of the ANX1 ectodomain grew at room temperature in hanging drops composed of 1.0 µl protein solution (25 mg ml^−1^) and 1.0 µl crystallization buffer [27%(*w*/*v*) PEG 3350, 0.1 *M* HEPES pH 7.5] suspended over 0.5 ml crystallization buffer. For structure solution, ANX1 crystals were transferred into crystallization buffer supplemented with 1 m*M* (NH_4_)_2_PtCl_4_ for 5 h, harvested, cryoprotected in crystallization buffer containing 15%(*v*/*v*) ethylene glycol and snap-cooled in liquid nitrogen. A 2.1 Å resolution data set was collected close to the Pt *L*_III_ edge (11566.3 eV, *f*′ = −16.8, *f*′′ = 12.2) on beamline PXIII at the Swiss Light Source (SLS), Villigen, Switzerland. As structure solution by multiple-wavelength anomalous dispersion (MAD) was unsuccessful, we next collected a 1.9 Å resolution isomorphous native data set for SIRAS (single isomorphous replacement with anomalous scattering) analysis. Finally, a non-isomorphous high-resolution native data set at 1.48 Å was recorded.

Crystals of ANX2 grew at room temperature from a solution at 20 mg ml^−1^ with 25%(*w*/*v*) PEG 3350, 0.1 *M* HEPES pH 7.5 and were cryoprotected by serial transfer into crystallization buffer containing a final concentration of 20%(*v*/*v*) ethylene glycol and snap-cooled in liquid nitrogen. A complete data set at 1.08 Å resolution was collected on beamline PXIII at SLS. Data processing and scaling were performed in *XDS* (v. June 2017; Kabsch, 2010 ▸).

### 2.3. Structure determination and refinement   

The structure of ANX1 was solved by the SIRAS method using a platinum derivative. Derivative and native data were scaled using *XPREP* (Bruker) and ten platinum sites were located in *SHELXD* (Sheldrick, 2015 ▸). Site refinement and phasing was performed in *SHARP* (Bricogne *et al.*, 2003 ▸) at 3.0 Å resolution followed by NCS averaging and density modification in *phenix.resolve* (Terwilliger, 2003 ▸) to 1.89 Å resolution. The density-modified map was used for automatic model building in *Buccaneer* (Cowtan, 2006 ▸). The resulting partial model was used to generate starting phases for *ARP*/*wARP*7 (Langer *et al.*, 2008 ▸), which was used for automatic model building at 1.48 Å resolution. The model contained two molecules in the asymmetric unit and was completed by alternating cycles of manual model correction in *Coot* (Emsley & Cowtan, 2004 ▸) and restrained TLS refinement in *REFMAC*5 (Murshudov *et al.*, 2011 ▸). The final ANX1 model was used to determine the structure of ANX2 by molecular replacement as implemented in *Phaser* (McCoy *et al.*, 2007 ▸). The ANX2 solution comprised one molecule in the asymmetric unit with an associated solvent content of ∼40%. The structure was completed by alternating cycles of manual rebuilding in *Coot* and restrained TLS refinement in *REFMAC*. Structure-validation checks were performed using *MolProbity* (Chen *et al.*, 2010 ▸) and structural diagrams were prepared with *PyMOL* (https://pymol.org/) or *CHIMERA* (Pettersen *et al.*, 2004 ▸).

### 2.4. Isothermal titration calorimetry (ITC)   

Experiments were performed at 25°C using a Nano ITC (TA Instruments, New Castle, USA) with a 1.0 ml standard cell and a 250 µl titration syringe. Proteins were gel-filtrated into ITC buffer (20 m*M* sodium citrate pH 5.0, 150 m*M* NaCl), and carbohydrates (maltose, isomaltose, cellobiose, heptomaltose, PGA, xylohexaose, cellohexaose, 1,5-α-l-arabinohexaose, mannose, galactose and 1,4-β-d-galactobiose) were dissolved in the same buffer. A typical experiment consisted of injecting 10 µl of the carbohydrate ligand (∼600 µ*M*) into 50 µ*M* ANX1 or HERK1 solution in the cell at 150 s intervals. The ITC data were corrected for the heat of dilution by subtracting the mixing enthalpies for titrant-solution injections into protein-free ITC buffer. Sugars were obtained from Sigma–Aldrich or Megazyme. Experiments were performed in triplicate and the data were analyzed using the *NanoAnalyze* program (v.3.5) as provided by the manufacturer.

### 2.5. Size-exclusion chromatography   

Analytical gel-filtration experiments were performed using a Superdex 200 Increase 10/300 GL column (GE Healthcare) pre-equilibrated in 20 m*M* citric acid pH 5.0, 100 m*M* NaCl. 100 µl of the isolated ANX1 (5 mg ml^−1^), ANX2 (4.5 mg ml^−1^), HERK1 (5 mg ml^−1^) and THE1 (5 mg ml^−1^) ectodomains were loaded sequentially onto the column and elution (at 0.7 ml min^−1^) was monitored by ultraviolet absorbance at 280 nm. The column was calibrated with a mixture of the high-molecular-weight (HMW) and low-molecular-weight (LMW) kits from GE Healthcare. The peak fractions were analyzed by SDS–PAGE.

## 3. Results   

### 3.1. Overall structures of the ANX1 and ANX2 ectodomains   

**Figure 1:**
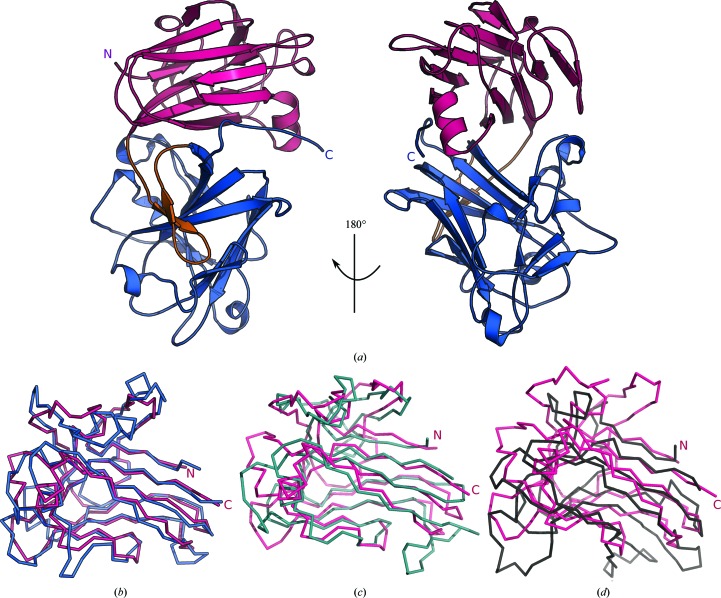
Architecture of the tandem malectin-like ectodomain of ANX1. (*a*) Front and 180° rotated views of the ANX1 ectodomain (ribbon diagram) with mal-N (residues 26–187) shown in magenta, the β-hairpin in orange and mal-C (residues 210–411) in blue. (*b*) Structural superposition of the ANX1 mal-­N domain (C^α^ trace; magenta) onto mal-C (blue). The r.m.s.d. is ∼2 Å comparing 127 corresponding C^α^ atoms. (*c*, *d*) Structural superpositions of the ANX1 mal-N domain (magenta) onto (*c*) the animal malectin from *X. laevis* (light blue; PDB entry 2k46; Schallus *et al.*, 2008 ▸) and (*d*) the bacterial carbohydrate-binding module (CBM22) from *Paenibacillus barcinonensis* (grey; PDB entry 4xur; Sainz-Polo *et al.*, 2015 ▸). The r.m.s.d.s are ∼2.2 Å comparing 127 corresponding C^α^ atoms and ∼3.1 Å comparing 119 corresponding C^α^ atoms, respectively.

**Figure 2:**
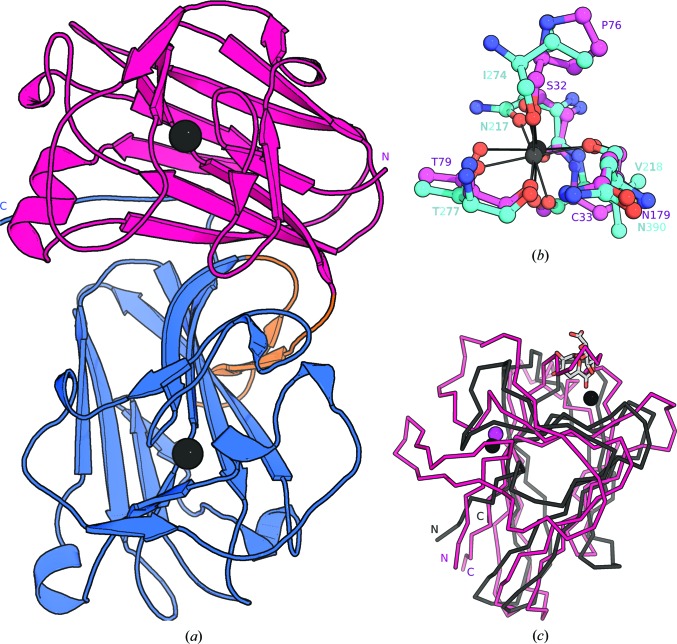
The malectin-like domains of ANX1 are structurally stabilized by calcium ions. (*a*) Ribbon diagram of the ANX1 ectodomain coloured as in Fig. 1 ▸ and including the positions of two stabilizing calcium ions (black spheres). (*b*) Details of the ANX1 mal-N (magenta) and mal-C (cyan) calcium ion-binding sites. Residues are in bond representation and metal-coordinating interactions are indicated by solid lines. (*c*) The ANX1 calcium-ion positions map to structural and not enzymatic calcium-binding sites in CBMs. Structural superposition of the ANX1 mal-N domain (ribbon diagram, in magenta) with the CBM35 bound to digalacturonic acid (PDB entry 2vzq; the r.m.s.d. is ∼3.7 Å comparing 68 corresponding C^α^ atoms; Montanier *et al.*, 2009 ▸) shown in grey. CBM35 contains two calcium ions, depicted as black spheres: one is in the catalytic binding site in close proximity to the carbohydrate and the other has a structural role and is located on the opposite face of the binding groove. The position of the latter site corresponds to the observed calcium-binding site in ANX1 (shown as a magenta sphere).

We produced the full-length ectodomains of *Arabidopsis* ANX1, ANX2, HERK1 and THE1 by secreted expression in insect cells and purified the N-glycosylated proteins to homogeneity (see §[Sec sec2]2). We obtained crystals for all receptors and diffraction-quality crystals for ANX1 and ANX2. We crystallized ANX1 in space group *P*1 (see Table 1 ▸) with two protein molecules per asymmetric unit. We determined the structure of ANX1 *via* single isomorphous replacement using a platinum derivative. ANX2 crystallized in space group *C*2, and the structure was solved by molecular replacement using the ANX1 structure as a search model (Table 1 ▸). The structures of ANX1 and ANX2 were refined to 1.48 and 1.1 Å resolution, respectively, with residues 26–411 in ANX1 and 28–411 in ANX2 being well defined in electron density. The ectodomains of ANX1 and ANX2 superimpose with an r.m.s.d. of ∼0.6 Å, comparing 375 corresponding C^α^ atoms in their ectodomains. ANX1 and ANX2 fold into two individual β-sandwich malectin-like domains consisting of four antiparallel β-strands connected by long loops and short helical segments (Fig. 1 ▸ *a*). The two domains pack tightly against each other, placing their β-sandwich cores at an angle of ∼85° (Fig. 1 ▸ *a*). A β-hairpin linker connects the N-terminal (mal-N) and the C-terminal (mal-C) malectin domains. The two malectin-like domains share an extensive hydrophilic interface with each other and with the β-hairpin linker (∼850 Å^2^ buried surface area; Fig. 1 ▸ *a*). The mal-N and mal-C domains align closely (r.m.s.d. of ∼1.5 Å for 138 corresponding C^α^ atoms; Fig. 1 ▸ *b*). The two highly conserved cysteine residues in *Cr*RLK1Ls are part of the structural cores of the mal-N and mal-C β-sandwich domains rather than being engaged in a disulfide bond (the S–S distance is ∼38.4 Å; Fig. 5*c*). A search with *DALI* (Holm & Sander, 1995 ▸) returned the animal malectin from *Xenopus laevis* as the closest structural homologue of ANX1 (*DALI* *Z*-score of 13, r.m.s.d. of ∼2.2 Å comparing 127 corresponding C^α^ atoms; Fig. 1 ▸ *c*; Schallus *et al.*, 2008 ▸). In addition, the ANX1 malectin domains share structural features with other carbohydrate-binding modules (CBMs) such as CBM22 and CBM35 from bacterial xylanases and hydrolases involved in plant cell-wall degradation (Figs. 1 ▸ *d* and 2 ▸; Sainz-Polo *et al.*, 2015 ▸; Montanier *et al.*, 2009 ▸). In mal-N and mal-C from ANX1 and ANX2 we located two calcium-binding sites which appear to function in structural stabilization rather than binding calcium as an enzymatic cofactor (Figs. 2 ▸ *a* and 2 ▸ *b*). Similar architectural calcium-binding sites have previously been described in other CBMs (Fig. 2 ▸ *c*; Boraston *et al.*, 2004 ▸).

### 3.2. Biochemical characteriz­ation of ANX1/2 tandem malectin-like domains   

**Figure 3:**
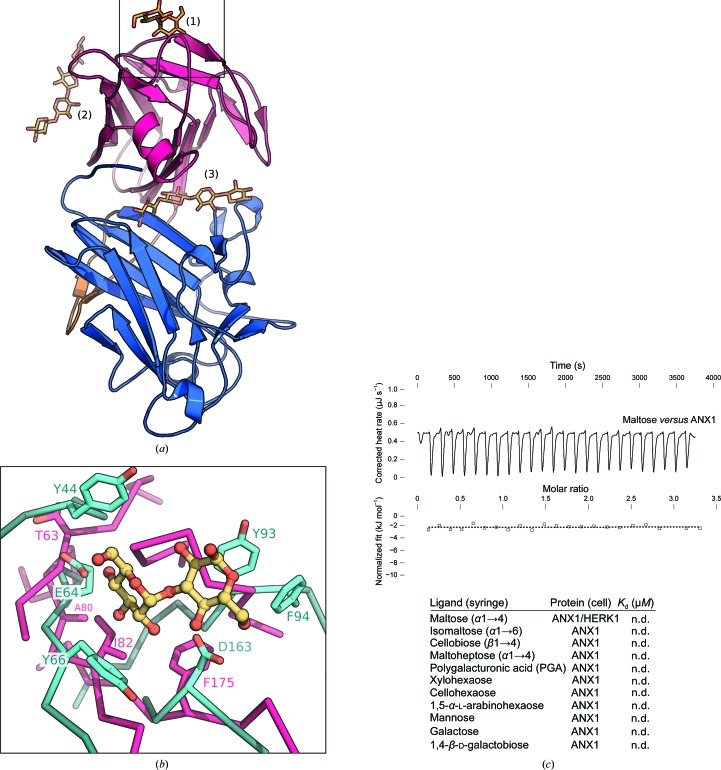
The malectin-like domains of ANX1 do not form canonical carbohydrate-binding sites. (*a*) Structural superposition of carbohydrate-binding sites from the malectin protein from *X. laevis* (PDB entry 2k46; Sainz-Polo *et al.*, 2015 ▸) and CBM22 from *P. barcinonensis* (PDB entry 4xur; Montanier *et al.*, 2009 ▸) onto ANX1 mal-N and mal-C (coloured as in Fig. 1 ▸). Carbohydrates are shown in bond representation (in yellow). (1) The nigerose-binding site of *X. laevis* malectin maps to the upper side of mal-N. (2) The binding surface of the xylotetraose in CBM22 is absent in mal-N; however, it maps to a potential binding cleft in ANX1 when superimposed on mal-C (3). (*b*) Close-up view of the nigerose-binding pocket of *X. laevis* malectin (shown in cyan) superimposed on ANX1 mal-N (magenta). The residues involved in nigerose binding are shown in cyan (in bond representation); the corresponding residues in ANX1 (magenta) are not conserved. (*c*) Isothermal titration calorimetry of d-maltose *versus* the ANX1 ectodomain; the table shows a summary for different carbohydrate polymers (*K*_d_, equilibrium dissociation constant; n.d., no detectable binding).

Animal malectins and the various CBMs have been shown to bind diverse carbohydrate ligands using different surface areas of their conserved β-sandwich ‘jelly-roll’ fold (Boraston *et al.*, 2004 ▸). As *Cr*RLK1Ls have been speculated to bind cell-wall components, we mapped the known carbohydrate-binding sites onto the ANX1 structure (Hématy *et al.*, 2007 ▸). Structural superposition of the *X. laevis* malectin bound to nigerose with mal-N from ANX1 identifies a potential carbohydrate-binding site on the external face of the N-terminal ANX1 β-sandwich domain (Fig. 3 ▸ *a*). While the overall arrangement of the secondary-structure elements in this region is conserved between *X. laevis* malectin and ANX1, the protruding loops and amino acids involved in carbohydrate binding are not present in the plant receptor ectodomain (Fig. 3 ▸ *b*; Schallus *et al.*, 2008 ▸, 2010 ▸). Consistently, we failed to detect interaction of the ANX1 ecto­domain with several glucose-derived disaccharides which had previously been shown to bind to the *X. laevis* malectin domain with micromolar affinity (Fig. 3 ▸ *c*). We next explored other potential carbohydrate-binding surfaces: Structural alignment of the bacterial CBM22 (*DALI Z*-score 8.6) with ANX1 mal-N brings the xylotetraose ligand of CBM22 into close proximity to the distal face of the β-sandwich domain normally used by B-type CBMs for the recognition of carbo­hydrate polymers. In ANX1, however, this surface area looks radically different from the known B-type CBMs (Fig. 3 ▸ *a*; Sainz-Polo *et al.*, 2015 ▸; Boraston *et al.*, 2004 ▸). Interestingly, the tetraose ligand maps to a cleft formed at the interface of mal-N and mal-C when CBM22 is superimposed onto mal-C (Fig. 3 ▸ *a*). We thus tested the binding of ANX1 to various cell-wall-derived carbohydrates of different natures and lengths (Park & Cosgrove, 2012 ▸; Pedersen *et al.*, 2012 ▸). However, we could not detect binding to any of the commercially available cell-wall polymers tested in isothermal titration calorimetry (ITC) assays (Fig. 3 ▸ *c*). Taken together, while the ANX1/2 malectin-like domains share extensive structural homology with both animal and bacterial carbohydrate-binding modules, the surface areas normally used to mediate the interaction with carbohydrate ligands appear not to be present in ANX1/2.

**Figure 4:**
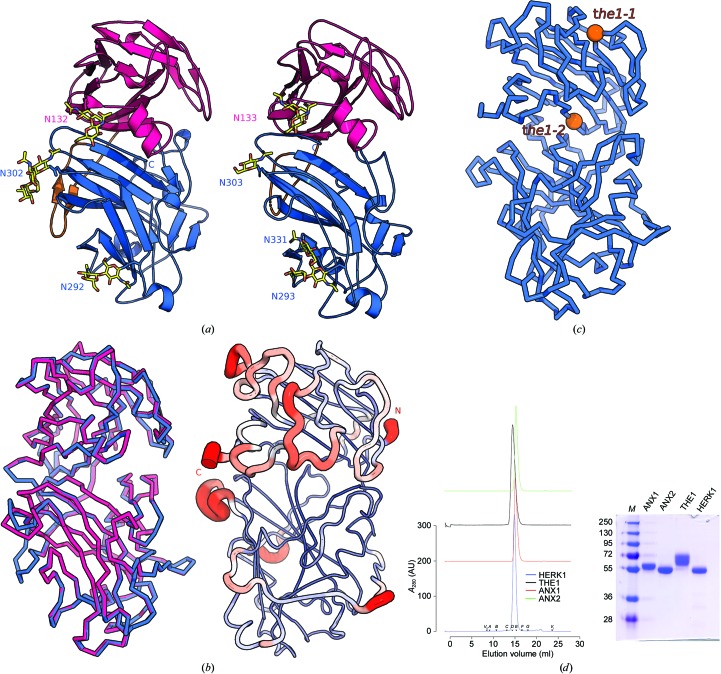
ANX1 and ANX2 share a common ectodomain architecture. (*a*) Ribbon diagrams of ANX1 (left) and ANX2 (right) show a strong degree of structural conservation (r.m.s.d. of ∼0.6 Å comparing 375 corresponding C^α^ atoms between ANX1 and ANX2) with a similar orientation of their mal-N and mal-C domains (colours are as in Fig. 1 ▸). The N-glycan structures observed in ANX1 and ANX2 are highlighted in yellow (in bond representation). (*b*) Structural superposition of the ANX1 (C^α^ trace, magenta) and ANX2 (blue) ectodomains (right) and a ribbon diagram of the ANX1 ectodomain with C^α^ atoms coloured according to their crystallographic temperature factors, from blue to red (left). Note that the N- and C-termini as well as several loop structures assembled around the ‘cleft’ region appear to be flexible. (*c*) The corresponding *the1-1* and *the1-2* alleles (shown as orange spheres) are mapped into the ANX1 structure. (*d*) Analytical size-exclusion chromatography reveals that the ANX1 extracellular domain elutes as a monomer (red line), as do the isolated THE1 (black line), HERK1 (blue line) and ANX2 (green line) ectodomains. The void volume (*V*_0_) and total volume (*V*_t_) are shown, together with elution volumes for molecular-mass standards (*A*, thyroglobulin, 669 000 Da; *B*, ferritin, 440 000 Da; *C*, aldolase, 158 000 Da; *D*, conalbumin, 75 000 Da; *E*, ovalbumin, 44 000 Da; *F*, carbonic anhydrase, 29 000 Da; *G*, ribonuclease A, 13 700 Da.). The molecular masses of purified ANX1, THE1 and HERK1 ectodomains analysed by MS-MALDI-TOF are 58, 63 and 53.5 kDa, respectively. An SDS–PAGE analysis of the purified ectodomains is shown alongside.

We next analyzed the glycosylation patterns of ANX1 and ANX2. We located three and four well defined N-glycans in our high-resolution ANX1 and ANX2 structures, respectively. In ANX1, glycans are positioned at Asn132 in mal-N and at Asn292 and Asn302 in mal-C (Figs. 4 ▸ *a* and 5 ▸ *c*). The glycosyl­ation pattern is conserved among ANX1 and ANX2, which harbours an additional site located at Asn331. The glycosyl­ation sites in our ANX1 and ANX2 structures map to the side and back of the tandem malectin assembly, leaving the mal-N–mal-C domain interface and surrounding loop regions accessible for potential interactions with ligands (Fig. 4 ▸ *a*). In line with this, analysis of the crystallographic temperature factors in ANX1 crystals reveal that several loop regions in mal-N are rather mobile (Fig. 4 ▸ *b*). It is of note that other carbohydrate-binding modules also make use of large flexible loops to form specific binding sites for carbohydrate ligands (Boraston *et al.*, 2004 ▸).

**Figure 5:**
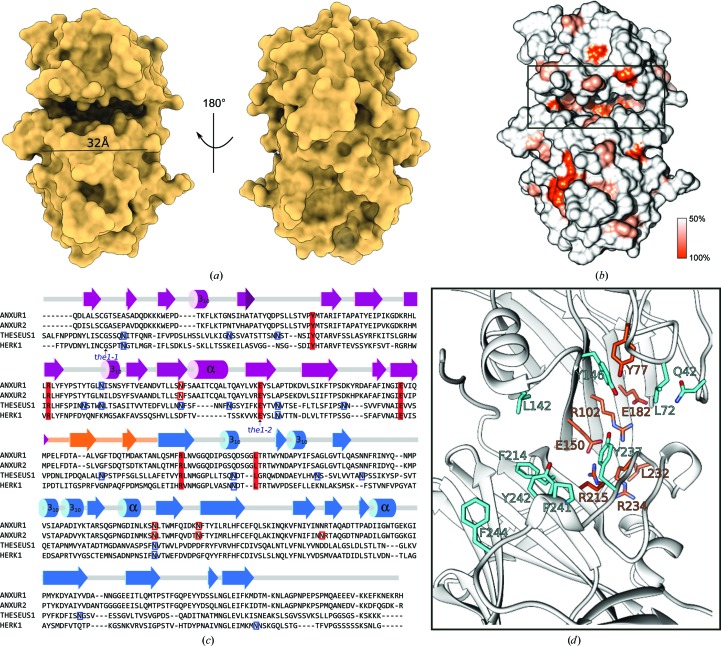
Plant tandem malectin-like receptor kinases feature a unique ligand-binding cleft. (*a*) Front and back views of the ANX1 ectodomain in surface representation reveal the presence of a wide and deep cleft located at the interface between the N- and C-terminal malectin-like domains. (*b*) Surface representation of ANX1 coloured according to *Cr*RLK1L family sequence conservation. (*c*) Sequence alignment with secondary-structure assignment for ANX1 calculated with *DSSP* (Touw *et al.*, 2015 ▸) and coloured according to Fig. 1 ▸. Predicted and experimentally verified N-glycosylation sites are highlighted in blue and red, respectively. The known genetic THE1 missense alleles are indicated by an arrow and the two conserved cysteine residues in *Cr*RLK1Ls are highlighted by light orange boxes. (*d*) Close-up view of ANX1 (as a ribbon diagram) with conserved interface residues highlighted in orange and with selected apolar and aromatic cleft-lining residues depicted in cyan (in bond representation). Residue identifiers are according to the ANX1 sequence. The depicted residues are highlighted in (*c*) using the same colour code.

Next, using a structure-based sequence alignment of ANX1 and the related *Cr*RLK1L THE1 involved in cell-wall sensing (Hématy *et al.*, 2007 ▸), we mapped the known genetic missense alleles of THE1 onto the ANX1 structure (Figs. 4 ▸ *c* and 5 ▸ *c*). The *the1-1* mutation (Gly37→Asp) and the *the1-2* allele (Glu150→Lys) are conserved among *Cr*RLK1L family members (Fig. 5 ▸ *c*). The *the1-1* mutation maps to the core of mal-N in ANX1/2, while the *the1-2* allele is located in the mal-N–mal-C domain interface (Fig. 4 ▸ *c*), suggesting that both mutations may interfere with the folding or structural integrity of the THE1 ectodomain, rationalizing their loss-of-function phenotypes (Hématy *et al.*, 2007 ▸).

Analysis of the lattice interactions in ANX1 crystals with *PISA* suggests that *Cr*RLK1L ectodomains may be monomers (Krissinel & Henrick, 2007 ▸; Table 1 ▸). Consistently, we found ANX1, ANX2 and the more distantly related *Cr*RLK1Ls HERK1 and THE1 to migrate as monomers in analytical size-exclusion chromatography experiments (Fig. 4 ▸ *d*).

We next sought to identify conserved interaction surfaces in the *Cr*RLK1L ectodomain by mapping a structure-based sequence alignment of the *Cr*RLK1L family onto the molecular structure of ANX1 (Fig. 5 ▸). We found that many highly conserved residues (shown in orange in Fig. 5 ▸ *b*) are located at the interface between mal-N and mal-C, contributing to the formation of a unique cleft structure which is about ∼30 Å in length and provides ∼1200 Å^2^ of accessible surface area (Fig. 5 ▸ *a*). Closer inspection of this cleft in ANX1 revealed the presence of several hydrophobic (Leu72, Leu142 and Pro241) and aromatic (Tyr146, Tyr214, Tyr237, Tyr242 and Phe244) amino acids exposed to the solvent (Figs. 5 ▸ *c* and 5 ▸ *d*).

## 4. Discussion   

Genetic studies have revealed important functions for the plant-unique *Cr*RLK1L membrane receptor kinases in very different physiological processes ranging from plant reproduction, cell elongation and growth to immunity (Li *et al.*, 2016 ▸; Nissen *et al.*, 2016 ▸). *Cr*RLK1Ls may regulate all of these different processes by controlling specific signalling events that lead to remodelling of the cell wall. Based on their distant sequence homology to animal carbohydrate-binding modules, the ectodomains of *Cr*RLK1Ls were originally proposed to bind carbohydrate ligands (Li *et al.*, 2016 ▸; Voxeur & Höfte, 2016 ▸). The structures of ANX1 and ANX2 reveal an un­expected novel fold with the two malectin-like domains packed against each other and connected by a short β-hairpin linker, forming a potential ligand-binding cleft. Similar results have recently been reported by Du *et al.* (2018 ▸) in an independent study. In the case of ANX1 the crystals from both studies were isomorphous (r.m.s.d. of 0.6 Å over 382 corresponding C^α^ atoms) and grew in similar conditions, with our crystals diffracting to higher resolution (1.48 Å). In our hands, the ANX2 receptor crystallized in a different crystal form and diffracted to 1.1 Å resolution. Comparison of the ANX2 structures resulted in an r.m.s.d. value of 0.5 Å over 377 corresponding C^α^ atoms.

Our structural comparison with known animal (Schallus *et al.*, 2008 ▸, 2010 ▸) and bacterial (Boraston *et al.*, 2004 ▸) carbo­hydrate-binding modules suggests that plant *Cr*RLK1Ls are noncanonical malectins or CBMs as they lack the conserved binding surfaces for carbohydrate ligands. It is of note that similar conclusions were drawn by Du and coworkers using a similar analysis. These structural observations are further supported by our biochemical assays; however, our experiments cannot rule out the possibility that plant *Cr*RLK1Ls may have evolved other, unique binding sites to sense complex plant cell-wall components that are not commercially available for biochemical studies. Indeed, the comparative structural analysis of different *Cr*RLK1Ls in these studies (this study and that of Du *et al.*, 2018 ▸) defined a cleft located at the interface between the N-terminal and C-terminal malectin-like domains, which based on its size, surface properties and sequence conservation could represent a bona fide binding site for a carbohydrate, a peptide or even a protein ligand. The speculation that this surface may be a ligand-binding site is supported by the lack of glycosylation in this region and the presence of flexible surface loops that differ in size and sequence among the different *Cr*RLK1L members.

Taken together, our structural analysis of the ANX1 and ANX2 fertilization receptors reveal a new tandem arrangement of two malectin-like domains which may have lost their ability to bind carbohydrates in a similar way to previously reported CBM binding surfaces and which form a new potential ligand-binding site located at the interface of the mal-N and mal-C domains. In line with this, it is of note that the plant *Cr*RLK1L FER has been shown to interact with secreted peptide hormones of the RALF family. Direct binding of RALF1 and RALF23 to FER has been reported using pull-down and ITC assays, with dissociation constants in the low micromolar range (Haruta *et al.*, 2014 ▸; Stegmann *et al.*, 2017 ▸). In the case of ANX1/2 and BUPS1/2, RALF4 and RALF19 have recently been proposed as ligands (Ge *et al.*, 2017 ▸). Alternatively, recently reported co-receptors, such as the *Cr*RLK1Ls BUPS1/2, members of the LRR-extensin protein family or receptor-like GPI-anchored proteins such as LORELEI, may be required for high-affinity RALF binding (Ge *et al.*, 2017 ▸; Li *et al.*, 2017 ▸; Mecchia *et al.*, 2017 ▸).

By defining the structural architecture of *Cr*RLK1L ectodomains, our work now sets the stage to biochemically identify and characterize the ligands of this important class of plant membrane receptors and to dissect their activation mechanism using structure–function approaches.

## Supplementary Material

PDB reference: ANXUR1, 6fig

PDB reference: ANXUR2, 6fih

## Figures and Tables

**Table 1:** Crystallographic data-collection and refinement statistics

	Anx1, (NH_4_)_2_PtCl_4_ derivative	Anx1, native 1	Anx1, native 2	Anx2, native
Data collection				
Space group	*P*1	*P*1	*P*1	*C*2
*a*, *b*, *c* (Å)	54.78, 68.55, 69.89	54.23, 68.50, 69.89	53.98, 68.74, 70.02	123.56, 41.76, 93.37
α, β, γ (°)	88.04, 75.57, 72.29	88.86, 74.71, 71.93	88.56, 74.97, 71.74	90, 117.4, 90
Resolution (Å)	48.39–2.13 (2.18–2.13)	48.39–1.89 (2.00–1.89)	47.27–1.48 (1.52–1.48)	44.29–1.08 (1.12–1.08)
*R*_meas_ †	0.082 (1.41)	0.112 (1.16)	0.062 (2.17)	0.041 (1.54)
CC_1/2_ † (%)	100 (67.6)	100 (50.5)	100 (68.0)	100 (47.1)
〈*I*/σ(*I*)〉†	10.7 (1.0)	6.9 (1.0)	17.9 (1.2)	18.98 (0.92)
Completeness† (%)	96.2 (81.5)	93.8 (93.4)	98.4 (95.6)	96.1 (80.54)
Redundancy†	3.5 (3.3)	1.8 (1.7)	10.3 (10.2)	6.5 (5.0)
Wilson *B* factor† (Å^2^)	51.0	35.8	31.4	14.70
Phasing				
Resolution‡ (Å)	48.39–3.00			
No. of sites‡	10			
Phasing power (iso)‡	0.449			
Phasing power (ano)‡	0.854			
FOM‡	0.344			
Refinement				
Resolution (Å)			47.27–1.48 (1.52–1.48)	44.29–1.08 (1.11–1.08)
No. of reflections			143968 (10394)	165089 (9560)
*R*_work_/*R*_free_ §			0.172/0.192 (0.303/0.309)	0.159/0.182 (0.470/0.535)
No. of atoms				
Protein			6130	3028
Glycan			164	95
Calcium			4	2
Water			743	433
Residual *B* factors§ (Å^2^)				
Protein			32.6	22.2
Glycan			51.2	37.7
Calcium			34.8	14.2
Water			41.8	39.3
R.m.s. deviations§				
Bond lengths (Å)			0.01	0.013
Bond angles (°)			1.47	1.70
*MolProbity* results				
Ramachandran outliers¶ (%)			0.1	0.0
Ramachandran favoured¶ (%)			97.0	98.93
*MolProbity* score¶			1.0	1.30
PDB code			6fig	6fih

† As defined in *XDS* (Kabsch, 2010 ▸).

‡ As defined in *SHARP* (Bricogne *et al.*, 2003 ▸).

§ As defined in *REFMAC*5 (Murshudov *et al.*, 2011 ▸).

¶ As defined in *MolProbity* (Chen *et al.*, 2010 ▸).
